# Impact of Barometric Pressure Changes on the Severity, Frequency, and Duration of Migraine Attacks: A Systematic Review of the Literature

**DOI:** 10.7759/cureus.96821

**Published:** 2025-11-14

**Authors:** Abduraheem Farah, Yusra Adam, Omer Ahmed, Reem Abdelhaleem Omar Ahmed, Mudather Abdelgabar Ali Mohammed, Rawan Awad Amer Ahmed, Ibrahim Mahfouz Osman Abdelrahman, Ahmed Mohamed Elamir, Ahmed Awad Amir Ahmed, Hisham Elnosh

**Affiliations:** 1 Anatomical Sciences, St. George's University, St. George, GRD; 2 Physiology, Neuroscience and Behavioral Sciences, St. George's University, St. George, GRD; 3 Internal Medicine, The National Ribat University, Khartoum, SDN; 4 Pathology, St. George's University, St. George, GRD; 5 Pathophysiology, St. George's University, St. George, GRD; 6 Microbiology Immunology and Pharmacology, St. George's University, St. George, GRD

**Keywords:** barometric pressure, environmental factors, headache disorders, migraine, systematic review

## Abstract

Migraine is one of the most prevalent neurological conditions, characterized by painful headache attacks accompanied by symptoms such as nausea, vomiting, photophobia, phonophobia, and sensory-visual disturbances. Multiple factors are considered potential triggers, including weather conditions. This review aims to evaluate and synthesize existing literature on the impact of barometric pressure changes on migraine severity, frequency, and duration.

This systematic review followed the Preferred Reporting Items for Systematic Reviews and Meta-Analyses (PRISMA) guidelines. After defining eligibility criteria, a comprehensive search was conducted in PubMed, SCOPUS, EMBASE, and CINAHL. Relevant studies were screened, and data were extracted using a predefined spreadsheet. Study quality and risk of bias were assessed using the NIH Quality Assessment Tool for Observational, Cohort, and Cross-Sectional Studies.

Of the 979 records identified, 14 (1.4%) studies met the inclusion criteria, comprising 2,696 participants aged 11 to 70 years. Most participants were female, 2,372 (87.9%). The majority of studies focused on adults and were conducted in geographically limited regions. All studies examined barometric pressure as the primary exposure, but methods for measuring pressure changes, assessing migraine severity, timing, and data sources varied substantially. Findings were inconsistent: several studies reported significant associations between pressure drops or rapid fluctuations and increased migraine frequency, fewer found associations with severity, and none identified a link with migraine duration.

Some evidence suggests a link between barometric pressure drops or fluctuations and increased migraine frequency. However, associations with migraine severity remain unclear, and no evidence supports a relationship with attack duration. The overall quality of existing evidence is limited by methodological weaknesses and potential biases, including heterogeneity in measurement methods, population characteristics, and study design. Further high-quality research, using standardized assessment tools and diverse, larger populations, is warranted to clarify the relationship between barometric pressure changes and migraine characteristics.

## Introduction and background

Headache is one of the most common medical conditions, with a prevalence of 90% among men and 95% in women [1]. Migraine is one of the most prevalent neurological conditions, causing painful headache attacks associated with a wide range of symptoms, including vomiting, nausea, photophobia, sensory-visual disturbances, and phonophobia [2,3]. It is a leading reason for neurological primary care visits and a disabling condition [4]. According to the International Headache Society (IHS), migraine can be classified into two main types: migraine with aura and migraine without aura, based on the presence or absence of an electrophysiological event known as aura [2]. Migraine can also be classified into episodic and chronic forms, and another severe and rare form, known as hemiplegic migraine, has also been identified [5].

Genetic factors can play a crucial role in defining susceptibility to migraine. It has been reported that a single gene polymorphism can lead to a condition known as monogenic migraine disorder [6]. Furthermore, heritability can account for 30% to 60% of migraine cases [7]. Additionally, 28 genetic loci have been found to be associated with headache, 14 (50%) of which have been linked with migraine [8]. Many variables can be considered triggering factors in migraine, most of which are associated with metabolic derangements. These include changes in sleep patterns, fasting, changes in ovarian hormone levels, alcohol, and physical exercise [9]. Other potential triggers can also include stress, perfume or odors, smoking, and sexual activity [10].

Several studies have explored the association between weather conditions and migraine, including humidity, temperature, rainfall, time zone, seasons, and others [11-14]. However, evidence on this association has been conflicting, especially regarding some weather parameters, such as barometric pressure. This systematic review aims to evaluate and synthesize the existing literature on the impact of barometric pressure changes on migraine severity, frequency, and duration, in order to better understand its potential role in migraine pathophysiology and inform future research and clinical practice.

## Review

Methods

This systematic review was performed according to the principles of the Preferred Reporting Items for Systematic Reviews and Meta-Analyses (PRISMA) [15]. The electronic database search was conducted on the following databases: PubMed, SCOPUS, EMBASE, and CINAHL. These databases were searched using the following keywords: Migraine, Migraine Disorders, Migraine Attack(s), Migraine Headache, Chronic Migraine, Episodic Migraine, Hemiplegic Migraine, Weather, Climate, Meteorological Factors, Atmospheric Pressure, Barometric Pressure, Air Pressure, Weather Change, Seasonal Variation, Environmental Factors, Severity, Intensity, Pain Score, Migraine Severity, Duration, Attack Duration, Headache Frequency, Recurrence, and Pain Measurement. A search strategy was used, combining these keywords with various Boolean operators, MeSH terms, and different database filtering methods. Additionally, reference lists of relevant reviews were searched manually for potentially eligible studies. We did not apply any restriction on the year of publication; however, we only considered records in the English language. These databases were searched up to March 2025.

Regarding the eligibility criteria, editorials, commentaries, letters, and conference proceedings were excluded from this review; otherwise, no restriction was applied in terms of study design. We included studies that investigated the impact of barometric pressure on migraine (no restriction was applied with regard to the type of migraine or the study population). The studies had to provide adequate data on the measurement methods used to assess barometric pressure and its impact on the duration, frequency, and severity of migraine attacks.

Following the removal of duplicated records, more than one reviewer was involved in the process of title and abstract screening to determine the potentially eligible studies; any discrepancies in inclusion or exclusion decisions were resolved by consensus. Full texts of the potentially eligible studies were assessed according to the predefined criteria mentioned above, and relevant studies were selected for inclusion. A spreadsheet was used to extract the relevant information from these included studies. The extracted data included information about the study aim, design, country, population demographics, sample size, type of barometric pressure change assessed in the studies (e.g., increase, decrease, and rapid fluctuation), method(s) of barometric pressure measurement (e.g., meteorological data and personal barometers), duration of exposure to barometric pressure considered (e.g., immediate, hours, and days before migraine onset), migraine severity measurement method (e.g., VAS, MIDAS score, and HIT-6), and change observed in migraine severity, frequency, and duration.

Regarding study quality and risk of bias assessment, it was performed by more than one reviewer using the NIH Quality Assessment Tool for Observational Cohort and Cross-Sectional Studies [16]. The overall quality ratings were as follows: Good - minimal risk of bias; all or nearly all criteria met. Fair - some risk of bias, but not enough to invalidate results. Poor - significant risk of bias that likely compromises the validity of findings.

Results

Study Selection and Characteristics of Included Studies

**Figure 1:**
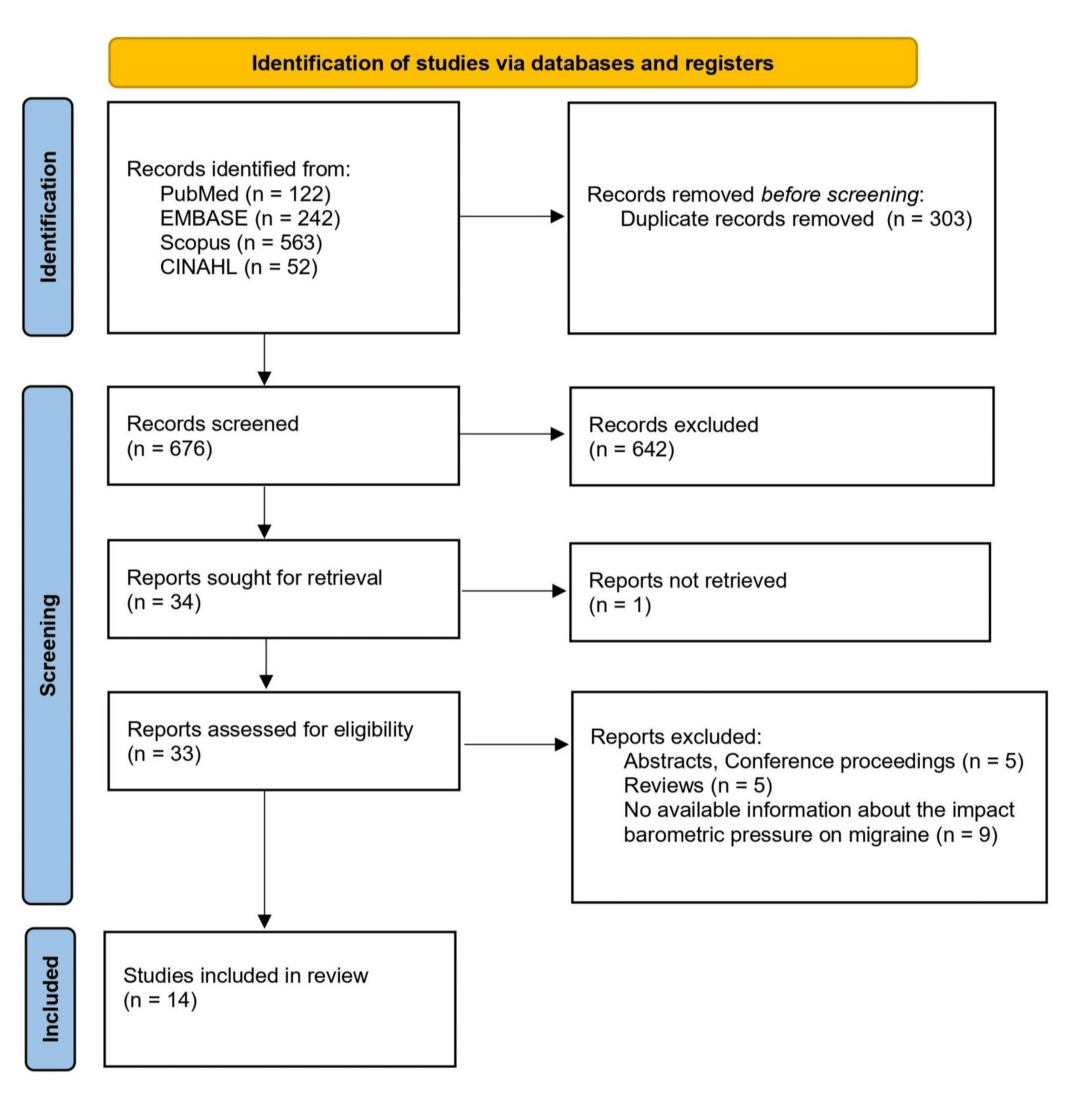
PRISMA flow chart of the study selection process PRISMA, Preferred Reporting Items for Systematic Reviews and Meta-Analyses

A total of 979 records were identified through the database search. Following title and abstract screening, 33 articles (3.4%) were assessed in full text against the predefined eligibility criteria. Of these, 14 studies (42.4%) met the inclusion criteria and were included in this systematic review. The detailed process of study selection is illustrated in Figure 1. Regarding the study design of the included studies, three of them were retrospective (21.4%), while the rest followed a prospective study design. Additionally, only one study (7.1%) was cross-sectional in nature, whereas the others were longitudinal studies. The basic characteristics of the included studies are detailed in Table 1.

**Table 1:** Basic characteristics of the included studies

Study ID	Study Design	Study Country	Study Aim	Study Settings	Sample Size	Age Group(s), Years	Gender (Male:Female)
								Gomersall and Stuart (1973) [17]	Observational, Longitudinal, Prospective study	United Kingdom	To examine the factors associated with migraine attacks, with a particular emphasis on identifying possible triggers such as weather conditions, psychological stress, and food or drink consumption.	Community-based (16 km radius)	56	Adults and Pediatrics (age range: 11-70)	12:44
Cull (1981) [18]	Observational, Longitudinal, Prospective study	United Kingdom	To examine the impact of spontaneous barometric pressure changes on a group of migraine sufferers visiting a hospital clinic. Additionally, seasonal and day-to-day variations in migraine attacks were analyzed.	Facility-based	44 (23 migraine without aura, 21 migraine with aura)	Adults and Pediatrics (age range: 16-55)	7:37
Casanova et al. (2022) [19]	Observational, Longitudinal, Prospective study	International	To examine the association between self-reported triggers and the onset of migraine attacks utilizing a smartphone application.	Community-based	328	Adults, mean: 42.3 (±12.9) years	38:290
Li et al. (2019) [20]	Observational, Longitudinal, Prospective study	United States of America	To investigate the relationships between barometric pressure and other factors and the onset of migraine headaches in patients with episodic migraine.	Facility-based	98	Adults, mean: 35 (±12) years	0.559722222
Katsuki et al. (2023) [21]	Observational, Cross-Sectional, Retrospective study	Japan	To examine the association between weather conditions and the frequency of headache occurrences by utilizing big data from a smartphone application and applying current statistical and deep learning methods.	Community-based	1627	Adults, mean: 34 (±11.2) years	176:1451
Hoffmann et al. (2011) [22]	Observational, Retrospective, Longitudinal, Case Series Analysis	Germany	To examine headache (including migraine) data over a 12-month period and relate these findings to specific weather components and their relative changes, with the aim of identifying any potential links to the occurrence or severity of migraine attacks.	Facility-based (patients’ residence: 50 km radius)	20 (migraine with aura n = 4, migraine without aura n = 16)	Adults (age range: 18-65)	5:15
Hoffmann et al. (2015) [23]	Observational, Retrospective, Longitudinal study	Germany	To examine whether a potential association between a specific meteorological condition and migraine could enable the individual prediction of a migraine attack.	Facility-based (patients’ residence: 50 km radius)	100	Adults (age range: 18-65)	NA
								Osterman et al. (1981) [24]	Observational, Longitudinal, Prospective study	Sweden	To examine the periodicity of migraine attacks, with a particular focus on identifying weekly patterns and exploring potential weather-related effects.	Community-based	53	Adults, median age: 40	12:41
								Kimoto et al. (2011) [25]	Observational, Longitudinal, Prospective study	Japan	To determine the impact of barometric pressure variations following weather changes on the occurrence of migraine attacks.	Facility-based (patients’ residence: 10 km radius)	28 (9, migraine with aura; 19, migraine without aura)	Adults, mean: 38	2:26
Akgün et al. (2021) [26]	Observational, Longitudinal, Prospective study	Turkey	To assess the impact of weather variables on the characteristics of episodic migraine and episodic tension-type headache attacks, including their frequency, duration, and severity.	Facility-based	50	Adults, mean: 37 (±11.3) years	18:32
								Zebenholzer et al. (2011) [27]	Observational, Longitudinal, Prospective study	Austria	To examine the association between variations in barometric pressure and the occurrence of migraines in individuals diagnosed with migraine.	Facility-based (patients’ residence: 25 km radius)	238	Adults, mean: 42.2 (±12)	29:209
																Villeneuve et al. (2006) [28]	Observational, Longitudinal, Prospective study	Canada	To examine the relationship between changes in barometric pressure and visits to the emergency department for migraineurs.	Facility-based (patients’ residence: 40 km radius)	4039 emergency department visits for migraines	Adults (age range: 20-40)	NA
								Cioffi et al. (2017) [29]	Observational, Longitudinal, Prospective study	Italy	To explore whether and how climate changes influence pain symptoms in migraineurs.	Facility-based	20	Adults, mean: 33.1 (±8.7) years	3:17
Okuma et al. (2015) [30]	Observational, Longitudinal, Prospective study	Japan	To examine the relationship between migraine attacks and changes in barometric pressure.	Facility-based	34 (22 had migraine with aura (MA), whereas 12 had migraine without aura (MOA))	Adults, mean: 32 (±6.7) years	3:31

Of the included studies, eight were conducted in Europe and three in Asia. Four studies were community-based, while the majority were facility-based. Regardless of setting, all studies were conducted in geographically restricted areas, with a radius of barometric pressure measurement ranging from 10 to 50 km. In total, 2,696 patients were included across the studies, of whom 2,279 (87.9%) were female. With the exception of two studies, all focused exclusively on adult populations. The age of participants ranged from 11 to 70 years.

Exposure to Barometric Pressure Changes and Migraine Data Measurement 

Barometric pressure was assessed as a primary exposure variable in all included studies; however, the directionality of pressure changes varied considerably. In the majority of the studies, the authors measured the impact of single weather values of barometric pressure, measured at a specific time, correlated with migraine attacks. Two of the studies assessed the impact of increasing and decreasing barometric pressure across the day on migraine [17,18]. Furthermore, in Okuma et al.’s study [30], a standardized value of barometric pressure was used as a reference, and the impact of deviations - either increases or decreases - from this baseline was evaluated. One study did not utilize objective barometric pressure measurements; instead, it relied on self-reported data through a structured questionnaire assessing perceived weather-related triggers, including barometric pressure. The assessed barometric pressure changes and corresponding measurement methods are summarized in Table 2 [19].

**Table 2:** Barometric pressure changes and corresponding measurement methods

Study ID	Barometric Pressure Change Assessed	Method of Barometric Pressure Measurement	Migraine Data Method of Measurement	Duration of Exposure to Barometric Pressure Considered	Migraine Severity Measurement Method
Gomersall and Stuart (1973) [17]	Rising fast, Rising slowly, Steady, Falling slowly, Falling fast barometric pressure	Meteorological data (midday measurement)	Self-reported data (patient charts)	Same day of the attack (immediate)	No standardized scale (mild or moderate and severe)
Cull (1981) [18]	Decreases in pressure (less than 1005 mb)/Increases in pressure (rise of more than 15 mb over preceding 24 hours)/Stable pressure (±5 mb changes)/Moderate decreases (falls between 6-15 mb over preceding 24 hours)	Meteorological data (at 06.00, 12.00, and 18.00 hours)	Self-reported data (a monthly record card)	Immediate (morning pressure), 6:00-18:00 hours, same day of the attack, and preceding 24-48 hours	No measurements for severity were recorded
Casanova et al. (2022) [19]	No weather values were entered	NA	Self-reported triggers questionnaire comparison	NA	No measurements for severity were recorded
Li et al. (2019) [20]	Single weather values	Meteorological data (recorded every 1-h intervals)	Self-reported data (headache diary)	Same day of the attack (immediate)	NA
Katsuki et al. (2023) [21]	Barometric differences across various times	Meteorological data (recorded hourly)	Self-reported data (electronic application, questionnaire)	6 hours before the attack to 2 days after	No measurements for severity were recorded
Hoffmann et al. (2011) [22]	Single weather values	Meteorological data (recorded every 4-h time frames)	Self-reported data (patient’s headache diary)	4 hours to 24 hours before the attack	No standardized scale (headache intensity is rated on a scale between 0 (no headache) and 5 (max. intensity))
Hoffmann et al. (2015) [23]	Single weather values	Meteorological data (recorded every 4-h time frames)	Self-reported data (headache diaries)	4 hours to 24 hours before the attack	No standardized scale (6-point Likert scale (0 = no headache, 5 = maximum intensity))
Osterman et al. (1981) [24]	NA	Meteorological data (recorded at 06:00, 14:00, and 22:00 hs)	Self-reported data (daily records using a special calendar)	Weekly	No measurements for severity were recorded
Kimoto et al. (2011) [25]	Single weather values	Meteorological data (recorded at 6:00 a.m., daily mean pressure and monthly mean pressure)	Mixed methods (headache diaries, medical records, structured clinical interview)	2 days before to 2 days after the headache	No measurements for severity were recorded
Akgün et al. (2021) [26]	Single weather values	Meteorological data (recorded at 12.00 noon every day)	Self-reported data (headache diary)	Same day of the attack (immediate)	NA
Zebenholzer et al. (2011) [27]	1 - single weather values, 2 - day-to-day changes in these values, 3 - a synoptic weather classification	Meteorological data (recorded every 10 mins)	Self-reported data (headache diary, questionnaire)	Same day of the attack (immediate)	No measurements for severity were recorded
Villeneuve et al. (2006) [28]	N/A	Meteorological data	Hospital records	Immediate, the preceding 24-48 hours	No measurements were recorded (the authors explicitly noted that ED visits likely reflect more severe forms of migraines)
Cioffi et al. (2017) [29]	Single weather values	Recorded via a weather sensor (custom-made portable data logger)	Self-reported data (custom-made portable data logger)	Same day of the attack (immediate)	10 cm electronic visual analogue scale (VAS)
Okuma et al. (2015) [30]	Deviations from the standard barometric pressure (increase or decrease)	Meteorological data, recorded by patients	Self-reported data (headache diary)	Same day of the attack (immediate)	No measurements for severity were recorded

The methods of barometric pressure measurement also differed across the included studies. Although most of the studies - with the exception of one study - relied on recordings of meteorological data, there was significant heterogeneity in the timing and frequency of pressure recordings [19]. Two studies used hourly recordings of barometric pressure [20,21], while two others employed four-hour time frames [22,23]. Three of the studies used morning pressure measured at 6:00 a.m. daily [18,24,25]. Additionally, four studies relied on midday recordings [17,18,24,26]. One study used high-frequency measurements at 10-minute intervals [27]. Meteorological data used in the included studies were also acquired from different sources, including barometric sensor stations, custom-made portable weather sensors, and self-reported data by the patients.

The duration of barometric pressure exposure varied across studies, ranging from same-day measurements associated with migraine onset to cumulative effects observed over a week. There were also notable disparities in migraine data used in the assessment. One study relied on hospital records to measure the relationship between changes in barometric pressure and visits to the emergency department for migraineurs [28]. Another employed a mixed-methods approach, combining headache diaries, medical records, and structured clinical interviews [25]. The remaining studies used self-reported data, mainly in the form of headache diaries. The severity of migraine attacks was assessed in only six of the included studies, and only one of them used a standardized scale (10 cm electronic VAS); the rest used non-standardized scales [29].

Impact on Migraine Frequency

The relationship between barometric pressure changes and migraine frequency was explored across several studies. In early studies, Gomersall and Stuart found no significant difference in the observed versus expected number of migraine attacks at different levels of atmospheric pressure. While fewer attacks were observed during low-pressure periods, this difference was not statistically significant [17]. In contrast, in studies conducted by Cull and Osterman et al., significant associations were found between barometric pressure changes and migraine frequency. Both studies reported a reduced frequency of migraines when the barometric pressure was below 1005 mb at 06:00 hours, compared to higher pressures ranging from 1016 to 1025 mb [18,24]. Additionally, a rise in pressure of more than 15 mb over 24 hours was linked to a significant reduction in migraine frequency. However, Osterman et al. also noted that no significant association was found between low barometric pressure and increased migraine frequency, contradicting some patient-reported beliefs [24].

More recent studies, such as Kimoto et al., demonstrated that a significant decrease in migraine frequency was associated with a rise in barometric pressure by more than 5 hPa from the day the headache occurred to two days after. Notably, individuals in the weather-sensitive group showed a significant increase in migraine frequency when the barometric pressure decreased by more than 5 hPa from the day of the headache to the following day [25]. On the other hand, Villeneuve et al., Akgün et al., and Li et al. observed no significant associations between barometric pressure and migraine frequency, including emergency department visits for migraine-related concerns [20,26,28]. Similarly, Zebenholzer et al. and Hoffmann et al. reported no significant associations between barometric pressure and migraine frequency [22,27]. Finally, Katsuki et al. identified an association between decreased barometric pressure six hours before a migraine and an increased hourly occurrence of headaches [21].

Impact on Migraine Severity and Duration

The association between barometric pressure changes and migraine severity has also been explored in several studies. Gomersall and Stuart found that a slightly higher percentage of severe migraine attacks (92.2%) occurred when barometric pressure was below 1020 mb, compared to mild or moderate attacks (87.2%). However, this difference was not statistically significant (p > 0.05) [17]. Hoffmann et al., on the other hand, observed a significant association between lower air pressure and increased migraine intensity, suggesting that reduced barometric pressure may contribute to more severe migraine attacks [22].

In contrast, another study that was also performed by Hoffmann et al. found no significant associations between weather variables, including barometric pressure, and migraine severity [23]. This aligns with other studies, like Li et al. and Akgün et al., where the associations between temperature, barometric pressure, and migraine onset were generally null [20,26]. Cioffi et al. found significant positive associations between VAS pain and temperature (p = 0.001) and atmospheric pressure (p = 0.027) in the migraine headache group [29].

The impact of barometric pressure changes on the duration of migraine attacks has been less frequently studied, in only three studies. Hoffmann et al. observed no significant associations between weather data, including barometric pressure, and the duration of migraine events. Similarly, Li et al. and Akgün et al. found no associations between temperature or barometric pressure and the duration of migraine attacks [20,23,26].

In contrast, another study that was also performed by Hoffmann et al. found no significant associations between weather variables, including barometric pressure, and migraine severity [23]. This aligns with other studies, like Li et al. and Akgün et al., where the associations between temperature, barometric pressure, and migraine onset were generally null [20,26]. Cioffi et al. found significant positive associations between VAS pain and temperature (p = 0.001) and atmospheric pressure (p = 0.027) in the migraine headache group [29].

The impact of barometric pressure changes on the duration of migraine attacks has been less frequently studied - in only three studies. Hoffmann et al. observed no significant associations between weather data, including barometric pressure, and the duration of migraine events. Similarly, Li et al. and Akgün et al. found no associations between temperature or barometric pressure and the duration of migraine attacks [20,23,26].

Methodological Quality and Risk of Bias Assessment

Using the NIH Quality Assessment Tool for Observational Cohort and Cross-Sectional Studies for the assessment of the risk of bias in this systematic review, overall, the quality of the studies was deemed fair (some risk of bias but not enough to invalidate results) in most of the studies, with the exception of one study, which was deemed of poor quality (significant risk of bias that likely compromises the validity of findings) [19].

All of the studies showed a high risk of bias in the domains of sample size justification and calculation. With the exception of one study, all of them had a high risk of bias in the domain of confounding factors adjustment [21]. One of the main limitations in the included studies was the reliance on self-reported data, which might be subject to reporting inaccuracies. Several studies did not use validated or standardized tools in the assessment of migraine severity, which might limit the comparability of outcomes [17,18,22,24,25,29]. Additionally, some studies were restricted by small sample sizes and overrepresentation of the female population, limiting the generalizability of their results [19,20,23,25,29,30].

Most studies were restricted to single regions or meteorological stations, and many of them did not account for seasonal variability or intra-day changes in atmospheric conditions [17,20,21,26-28]. Furthermore, the short duration of some of the included studies may have restricted the ability to detect long-term associations [24,26]. Moreover, another issue was analyzing data without accounting for inter-individual variability in weather sensitivity [17,18,21].

Regarding potential sources of bias in the included studies, recall bias was prevalent, especially in the studies relying on retrospective self-reporting [19,22,25,26,29,30]. Selection bias was also identified in several studies due to small, non-representative, or convenience samples [17,19,21,23,28]. Other potential sources of bias include weather forecast bias and exposure misclassification bias (Table 3).

**Table 3:** Risk of bias assessment ✔: low risk of bias, ❌: high risk of bias, ❓: unclear risk of bias, N/A: not applicable. 1 - Was the research question or objective clearly stated?, 2 - Was the study population clearly specified and defined?, 3 - Was the participation rate of eligible persons at least 50%?, 4 - Were all the subjects selected or recruited from the same or similar populations (including the same time period)?, 5 - Was a sample size justification, power description, or variance and effect estimates provided?, 6 - For the analyses, were the exposure(s) measured prior to the outcome(s)?, 7 - Was the timeframe sufficient to reasonably expect an association?, 8 - Were the exposure measures clearly defined, valid, reliable, and consistently implemented?, 9 - Were the exposure(s) assessed more than once over time?, 10 - Were the outcome measures clearly defined, valid, reliable, and consistently implemented?, 11 - Were the outcome assessors blinded to the exposure status?, 12 - Was the loss to follow-up after baseline 20% or less?, 13 - Were key potential confounding variables measured and adjusted statistically?, 14 - Were outcome measures taken multiple times to ensure consistency?

Study	1	2	3	4	5	6	7	8	9	10	11	12	13	14	Summary Quality
Gomersall and Stuart (1973) [17]	✔	✔	❓	✔	❌	✔	✔	✔	✔	✔	❌	✔	❌	✔	Fair
Cull (1981) [18]	✔	✔	❓	✔	❌	✔	✔	✔	✔	✔	❌	✔	❌	✔	Fair
Casanova et al. (2022) [19]	✔	❓	N/A	❌	❌	✔	✔	❓	❌	❓	N/A	N/A	❌	❌	Poor
Li et al. (2019) [20]	✔	❓	N/A	✔	❌	✔	✔	✔	✔	❓	N/A	N/A	❌	✔	Fair
Katsuki et al. (2023) [21]	✔	❓	N/A	✔	❌	✔	✔	✔	✔	❓	N/A	N/A	✔	✔	Fair
Hoffmann et al. (2011) [22]	✔	❓	N/A	✔	❌	✔	✔	✔	✔	❓	N/A	N/A	❌	✔	Fair
Hoffmann et al. (2015) [23]	✔	❓	N/A	✔	❌	✔	✔	✔	✔	❓	N/A	N/A	❌	✔	Fair
Osterman et al. (1981) [24]	✔	✔	❓	✔	❌	✔	❌	✔	✔	✔	❌	✔	❌	✔	Fair
Kimoto et al. (2011) [25]	✔	❓	N/A	✔	❌	✔	✔	✔	✔	❓	N/A	N/A	❌	✔	Fair
Akgün et al. (2021) [26]	✔	❓	N/A	✔	❌	✔	❌	✔	✔	❓	N/A	N/A	❌	✔	Fair
Zebenholzer et al. (2011) [27]	✔	❓	N/A	✔	❌	✔	✔	✔	✔	❓	N/A	N/A	❌	✔	Fair
Villeneuve et al. (2006) [28]	✔	❓	N/A	✔	❌	✔	✔	✔	✔	❓	N/A	N/A	❌	❓	Fair
Cioffi et al. (2017) [29]	✔	❓	N/A	✔	❌	✔	✔	✔	✔	❓	N/A	N/A	❌	✔	Fair
Okuma et al. (2015) [30]	✔	❓	N/A	✔	❌	✔	✔	✔	✔	❓	N/A	N/A	❌	✔	Fair

Discussion

The evidence from the included studies presents a suggestive relationship between barometric pressure changes and migraine attacks. While several studies reported significant associations - particularly linking pressure drops or rapid changes with increased migraine frequency - others found no significant association, highlighting inconsistency across the literature. Findings on migraine severity were also variable, with only a few studies demonstrating a significant relationship, and most relying on non-standardized tools for migraine severity assessment. The effect of barometric pressure on migraine duration was explored in only three studies, all of which found no significant association. Overall, the variability in study designs, barometric pressure measurements, and outcome assessment measures limits definitive conclusions. However, the collective findings suggest that barometric pressure fluctuations may act as a trigger, particularly in relation to migraine frequency.

Several theories have been proposed to explain how barometric pressure changes may affect headache. One theory suggests that stimulation of the sympathetic nervous system and adrenal medullary hormones in response to falling pressure can lead to tissue ischemia, peripheral vasoconstriction, and lower blood pH, all of which may contribute to headache onset [31,32]. Another theory proposes that decreased pressure increases discharge rates in the spinal trigeminal nucleus - especially in neurons receiving input from the dura mater and cornea - indicating possible activation of nociceptive pathways through structures such as the frontal sinus or inner ear [33]. A third hypothesis posits that alterations in sinus pressure during atmospheric pressure changes may trigger headaches through mechanisms similar to paranasal barotrauma, particularly in individuals with sinus structural abnormalities [34]. Additionally, reduced oxygen saturation at lower pressures may cause cerebral vasodilation and activation of the trigeminovascular system, potentially resulting in both vasogenic and cytotoxic edema [35,36].

Other weather parameters have also been found to impact migraine attacks. In a study conducted by Wang et al., 32.7% of migraine patients identified sunlight as a triggering factor, and 31.1% reported weather changes as a trigger - occurring more frequently among female patients compared to males [37]. In addition, a retrospective study by Yilmaz et al. found that migraine-related emergency department visits increased during periods of high temperature and low humidity; however, no significant association was found between migraine occurrence and lunar phases [38]. Other studies also reported that higher daily temperatures were associated with an increased number of migraine-related emergency department visits [39,40]. One study observed that weather-sensitive migraine patients were particularly affected by changes in temperature and humidity, and that some individuals were triggered by multiple environmental factors, with no significant differences found across sex, age, or specific weather conditions in subgroup analyses [41]. Moreover, some evidence suggests a significant association between migraine onset during the cold season and exposure to traffic-related air pollutants, such as NO_2_, O_3_, and CO [42-44].

In patients living at high latitudes, such as those in the Arctic Circle, seasonal changes and variations in sunlight exposure have also been suggested as potential migraine triggers. A study conducted among residents of Northern Norway found that migraine patients reported a higher frequency of attacks during the summer months, while non-migraine headaches were more prevalent during the darker winter season [14]. Another study suggested that sunny weather may act as a migraine trigger [45]. Patients with migraine generally have lower thresholds of discomfort to stimuli such as sound, light, and thermal or mechanical stimulation [46]. One study found that individuals with migraine tend to have higher sensitivity to light, particularly at low and high wavelengths [47]. It was also found that patients exhibit heightened sensitivity to blue and red light spectra, with increased light intensity linked to a greater migraine burden [48].

Several limitations should be considered when interpreting the findings of this systematic review. Many of the included studies used self-reported headache diaries and lacked standardized measures of migraine severity. Additionally, small sample sizes, limited geographic scope, and short observation periods were common, which may restrict generalizability. There was considerable variability in how barometric pressure was recorded (e.g., frequency, timing, and measurement source), limiting comparability across studies. Moreover, potential sources of bias, such as recall bias, selection bias, and lack of adjustment for key confounders - including other weather parameters and patient demographics - were evident across most of the studies. Failure to account for individual variability in weather sensitivity was also a recurrent limitation.

## Conclusions

The collective findings drawn from the included studies suggest that barometric pressure fluctuations may act as a trigger, particularly in relation to migraine frequency. Several studies reported significant associations, particularly associating pressure drops or rapid changes with increased migraine frequency; however, few studies demonstrated a significant association between barometric pressure and migraine severity, and no association was found in relation to migraine duration. While the evidence suggests a possible relationship between barometric pressure changes and migraine characteristics, the overall quality of available studies is limited by methodological shortcomings and potential biases. Future research should address these limitations through larger, more diverse samples, standardized outcome measures, and better control for confounding variables, to clarify this association.
